# Predictors of Acute Renal Injury Study (PARIS) among HIV-positive individuals: design and methods

**DOI:** 10.1186/s12882-017-0696-1

**Published:** 2017-09-07

**Authors:** Celia P. Corona-Villalobos, Michael G. Shlipak, Adrienne Tin, Chirag Parikh, Richard D. Moore, Eric Vittinghoff, Jose Manuel Monroy-Trujillo, Mohamed G. Atta, Michelle M. Estrella

**Affiliations:** 1 Department of Medicine, Johns Hopkins School of Medicine, 1830 E. Monument St., Suite 416, Baltimore, 21287 MD USA; 2 San Francisco and San Francisco VA Health Care System, Kidney Health Research Collaborative, University of California, 1450 Clement St., 111A1, San Francisco, 94121 CA USA; 3 Department of Epidemiology, Johns Hopkins Bloomberg School of Public Health, 615 N. Wolfe St., Room W6017, Baltimore, 21287 MD USA; 4 Department of Medicine, Division of Nephrology, Yale University School of Medicine, 60 Temple St., Suite 6C, New Haven, 06510 CT USA; 5 Department of Medicine, Division of General Internal Medicine, Johns Hopkins School of Medicine, 1830 E. Monument St., Suite 8059, Baltimore, 21287 MD USA

## Abstract

**Background:**

Acute kidney injury (AKI), which is common among HIV-positive individuals, may contribute to the excess burden of chronic kidney disease (CKD) in this patient population; however, conventional clinical methods to detect AKI do not capture kidney injury sufficiently early to prevent irreversible damage. Further, large observational and interventional studies of AKI generally exclude HIV-positive persons in spite of their disproportionate risk.

**Methods:**

The Predictors of Acute Renal Injury Study (PARIS) is a prospective observational cohort study among HIV-positive individuals established to determine the ability of candidate kidney injury biomarkers to predict future hospitalized clinical AKI, to characterize hospitalized subclinical AKI, and to discern the risk of progressive kidney disease following subclinical and clinical AKI. Among the candidate kidney injury markers, we will select the most promising to translate into a clinically viable, multiplex panel of urinary biomarkers which we will integrate with clinical factors to develop a model prognostic of risks for AKI and subsequent kidney function decline. This study has a targeted enrollment of 2000 participants. The overall follow-up of participants consists of two phases: 1) a 5-year active follow-up phase which involves serial evaluations at enrollment, annual clinic visits, and among participants who are hospitalized during this period, an evaluation at index hospitalization and 3 and 12 months post-hospitalization; and 2) a subsequent passive follow-up phase for the duration that the participant receives medical care at The Johns Hopkins Hospital.

**Discussions:**

This study will serve as an important resource for future studies of AKI by establishing a repository with both ambulatory and inpatient biospecimens, a resource that is currently lacking in existing HIV clinical cohorts and studies of AKI. Upon completion of this study, the resulting prognostic model which will incorporate results from the multiplex HIV-AKI Risk Pane could serve as a pharmacodynamic endpoint for early phase therapeutic candidates for AKI.

## Background

In the modern era of effective antiretroviral therapy, HIV-positive individuals remain at increased risk of developing chronic kidney disease (CKD) and progressing to end-stage renal disease (ESRD). This risk remains substantial even among persons who achieve adequate viral suppression and is not fully explained by traditional risk factors for CKD. Acute kidney injury (AKI), which affects approximately 1 in 6 hospitalized HIV-positive patients, may be contributing to this persistent CKD burden [1].

Studies in the general population indicate that clinical repercussions of AKI persist beyond hospitalizations, even as long as 10 years after AKI has occurred [2]. Among HIV-positive patients who survive at least 90 days after their hospitalization, AKI severity has been associated with a 2- to 4-fold increased risk of heart failure, a 1.3- to 2-fold increased risk of cardiovascular disease, a 3.8- to 20-fold increased risk of ESRD and up to a 1.7-fold increased risk of all-cause mortality [1]. Even among patients who return to their baseline serum creatinine, the risk of progression to ESRD and death remain significantly elevated. These findings suggest that HIV-positive individuals may sustain extensive kidney injury that is not fully captured by currently available clinical methods, such as serum creatinine, to detect AKI and measure its severity.

Although the current national guidelines define AKI based on absolute and relative increases in serum creatinine and/ or declines in urine output, these parameters may not be altered until over half of kidney function has been lost. Given these limitations, many individuals may sustain critical kidney damage despite serum creatinine levels and urine output remaining unchanged. This condition has been described as “subclinical AKI” [3] characterized by elevations in urine biomarkers of kidney injury, such as kidney injury marker-1 (KIM-1) and preserved serum creatinine levels. Recently, the Translational Research Investigating Biomarker Endpoints in AKI (TRIBE-AKI) Consortium has shown that among patients with clinical AKI following major cardiac surgery [4], the highest compared to the lowest tertiles of urine neutrophil gelatinase-associated lipocalin (NGAL), IL-18 and KIM-1 were each independently associated with a more than doubling of mortality risk after hospital discharge [5]. Patients with subclinical AKI (defined by urine IL-18 and KIM-1 elevations in the absence of serum creatinine elevations) also had significantly higher 3-year mortality risk after hospitalization. Urine biomarkers, which are sensitive and specific for renal tubulointerstitial injury, inflammation and fibrosis, are growing in number and reshaping the concept of AKI into a continuum of risk [6] which spans from “at-risk” populations, to subclinical AKI to clinically overt AKI. However, the current understanding of hospitalized AKI has primarily relied on serum creatinine levels at hospital admission. Furthermore, the prognostic and predictive performance of novel urine biomarkers of kidney injury has largely been assessed without baseline ambulatory levels due to the lack of stored blood and urine samples prior to AKI events in most if not all studies [7–10]. Most importantly, despite the burden of AKI in the HIV-positive population and recent advances in kidney injury biomarkers, very few studies of AKI have comprehensively focused on this high-risk patient population. Consequently, there remains a lack of strategies to identify HIV-positive persons at highest risk of AKI and its related adverse outcomes. We hypothesize that higher ambulatory levels of urine biomarkers denote ongoing subclinical kidney injury that portends higher risk of subsequent hospitalization and clinical AKI. We further postulate that higher urine biomarker levels of kidney injury during hospitalization are predictive of longitudinal kidney function decline.

To address these unmet clinical needs and ultimately mitigate the disproportionate risk of AKI and its consequences in the HIV-positive population, we established the Predictors of Acute Renal Injury Study (PARIS) in 2015. Through support from the National Institute of Diabetes, Digestive and Kidney Diseases (R01-DK-103574) and additional resources from the Johns Hopkins Institute of Clinical and Translational Research (ICTR) and Center for AIDS Research (1P30-AI-094189), this study will determine the ability of candidate kidney injury biomarkers to predict hospitalized clinical AKI, to characterize subclinical AKI, and to discern the risk of progressive kidney disease following subclinical and clinical AKI among a prospective cohort of HIV-positive individuals. PARIS specifically aims to:

- Establish a prospective cohort of HIV-positive individuals treated in a large academic HIV practice, the Johns Hopkins Hospital HIV Clinic, with detailed clinical data and biological samples collected in both the outpatient and inpatient settings
- Determine the association of ambulatory kidney damage with incident hospitalized clinical AKI and progressive kidney disease after AKI
- Investigate the prevalence of subclinical and clinical AKI among hospitalized HIV-positive individuals and their associations with progressive kidney disease after hospitalization
- Develop a prognostic model that integrates a multiplex panel of complementary urine biomarkers and clinical variables that will distinguish risk for incident AKI and subsequent progressive kidney disease and that will be feasible to implement in clinical practice

The culmination of this work will greatly enhance our understanding of subclinical and clinical AKI and their contribution to adverse health outcomes among HIV-positive persons. In addition, it will yield a prognostic model that incorporates a clinically applicable multiplex HIV-AKI Risk Panel which could then be tested as a screening tool in clinical trials of AKI management as well as a pharmacodynamic endpoint for early phase therapeutic candidates for AKI [11].

## Methods

### Overall study design

PARIS is a collaboration among investigators at the University of California, San Francisco, San Francisco Veterans Affairs Medical Center, Yale University and Johns Hopkins School of Medicine. This prospective observational cohort study enrolls and follows all eligible HIV-positive individuals who are receiving care through the Johns Hopkins Hospital HIV Clinic in Baltimore, Maryland, USA. To augment data collection, PARIS will leverage the established infrastructure of the Johns Hopkins HIV Clinical Cohort (JHHCC), an NIH-sponsored open cohort established in 1990 to provide longitudinal data on HIV-positive patients cared for at the Moore Clinic [12]. The JHHCC enrolls new patients as they initiate longitudinal HIV care at the HIV Clinic, with a consent rate of over 98%. The Institutional Review Boards at the University of California, San Francisco, the San Francisco Veterans Affairs Medical Center, Yale University, and the Johns Hopkins School of Medicine approved the study.

**Fig. 1:**
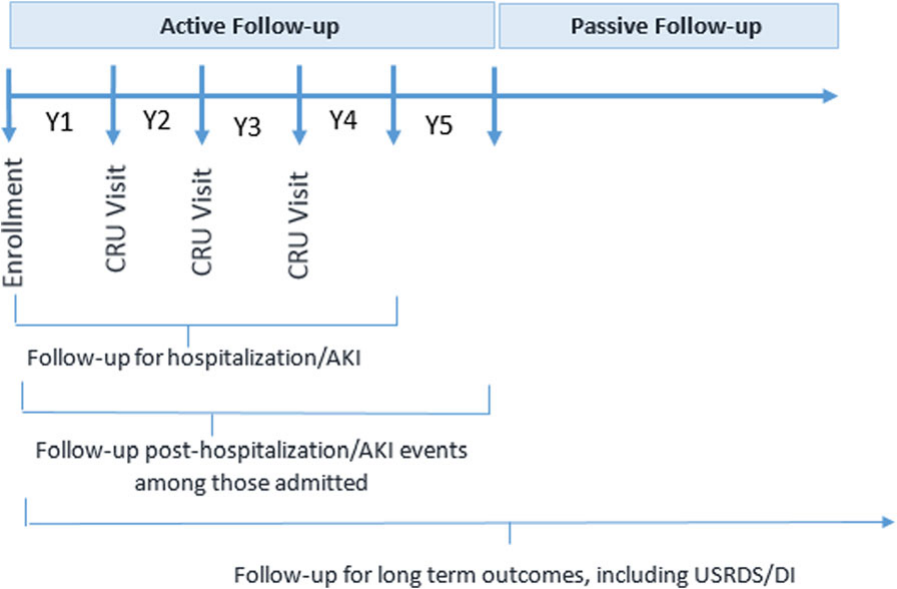
Phases of Follow-up in the PARIS Cohort. The active follow-up phase consists of 3 years of annual ambulatory follow-up visits for questionnaires and biospecimen collection and up to 4 years from enrollment for capture of hospitalization and AKI events among all participants, and an additional year of follow-up among those who are hospitalized. The subsequent passive follow-up phase allows for ongoing evaluation of long-term outcomes for the duration that the participant receives medical care within the Johns Hopkins Health System. AKI: Acute Kidney Injury; CRU: Clinical Research Unit, USRDS: United States Renal Data System, DI: Death Index

The overall follow-up of participants consists of two phases: 1) a 5-year active follow-up phase; and 2) a subsequent passive follow-up phase for the duration that the participant receives medical care within the Johns Hopkins Health System. The active follow-up phase enables investigators to prospectively collect data and biospecimens from the participants at scheduled study visits and during hospitalized clinical events of interest. This phase is comprised of a baseline visit and 3 years of annual ambulatory follow-up visits in the Johns Hopkins Outpatient Clinical Research Unit among all participants for questionnaires and biospecimen collection**,** up to 4 years from enrollment for capture of hospitalization and AKI events among all participants, and an additional year of follow-up among those who are hospitalized. The subsequent passive follow-up phase allows for ongoing evaluation of long-term outcomes, such as cardiovascular disease events, ESRD, and mortality. This phase entails ongoing review of the participants’ health records as well as linkage to outcomes registries, such as the U.S. Renal Data System and the National Death Index (Fig. 1).

**Fig. 2:**
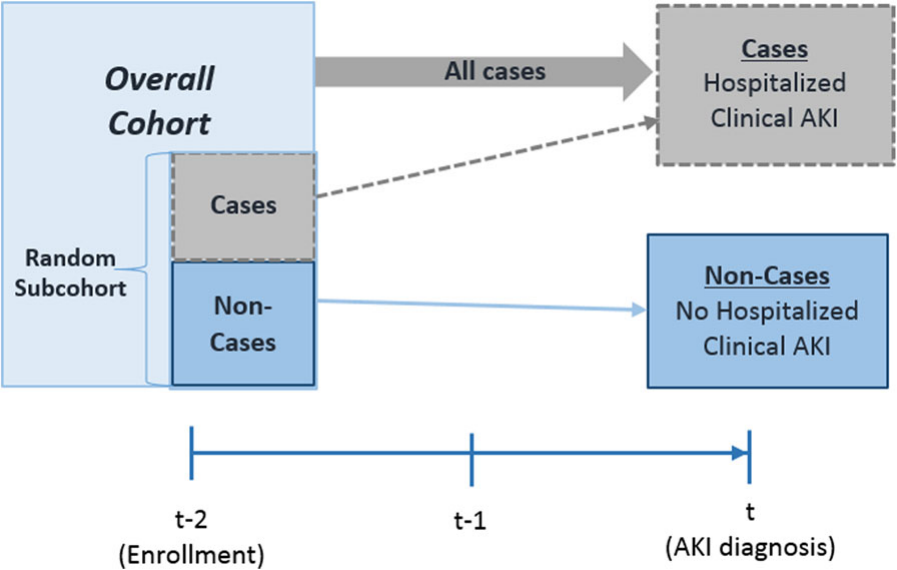
Nested Case-Cohort Design for Annual Ambulatory Biomarker Measurements. In the nested case-cohort design, annual ambulatory biomarkers will be measured only among incident cases of hospitalized clinical AKI and a random subsample of the overall cohort. AKI: Acute Kidney Injury; t: Time

While biospecimens are collected on all participants, a nested case-cohort design [13] will be employed to evaluate the association between ambulatory levels of urine biomarkers with incident clinical AKI. In the nested case-cohort, annual ambulatory biomarkers will be measured only among incident cases of hospitalized clinical AKI and a random sample of the overall population (Fig. 2). This approach enables estimation of risks without having to measure biomarkers repeatedly on all participants and yields unbiased estimates of the population prevalence of AKI risk factors [13]. It also provides a subcohort representative of the overall population that allows study of other outcomes. To investigate the prevalence of subclinical and clinical AKI among hospitalized HIV-positive individuals and their associations with progressive kidney disease after hospitalization, a traditional cohort design will be used.

### Cohort participants

#### Source population

The PARIS Study enrolls among HIV-positive patients who receive ambulatory care at the Johns Hopkins HIV Clinic. This clinic represents the largest program for HIV care within the state of Maryland, USA, currently with over 2800 HIV-positive patients and approximately 20,000 patient-visits annually. It provides multidisciplinary care tailored to the needs of patients with HIV. Based on data captured by the JHHCC, approximately 77% and 3% of the patients self-report as African American and Latino, respectively, and 35% are women. The current median age is 48 years (interquartile range [IQR]: 40 to 54 years). Approximately 27% report homosexual activity while 33% report intravenous drug use. Thirty-seven percent are co-infected with the hepatitis C virus. At enrollment, the median estimated glomerular filtration rate (eGFR) is 105.0 mL/min/1.73 m^2^ (IQR: 87.0–123.0). More than 90% of ambulatory and inpatient care for Moore Clinic patients is delivered within The Johns Hopkins Hospital. In addition, 98.4% of patients who receive care in the Moore Clinic are also enrolled in the JHHCC.

#### Participant selection

To participate in PARIS, a patient must meet the following inclusion criteria: 1) HIV-positive; 2) age 18 years or older; 3) English-speaking; 4) actively followed clinically at the Johns Hopkins HIV Clinic; and 5) has not experienced AKI within the preceding 6 months. Patients who have experienced clinical AKI within the preceding 6 months; reside in a hospice, skilled nursing facility or prison; have health conditions that interfere with study participation (e.g. significant cognitive impairment or active psychotic illness); plan to move out of state within the next year; are receiving chronic renal replacement therapy, have an eGFR of <15 mL/min/1.73m^2^; have had a previous solid organ transplant; or self-report current pregnancy are excluded. To ascertain potential participants’ eligibility on these criteria, we conduct patient interviews and review electronic health records. The participant’s most recent value recorded in the electronic health record within the preceding year is regarded as the baseline serum creatinine. The baseline eGFR is calculated using either the abbreviated Modification of Diet in Renal Disease (MDRD) [14] as used within the Johns Hopkins Medicine Pathology Department or the CKD-Epidemiology Collaboration (CKD-EPI) equation [15] as used by Quest Diagnostics (Quest Diagnostics, Inc., Madison, MJ, USA) and LabCorp (LabCorp Diagnostics, Burlington, NC, USA). All clinical serum creatinine results are measured using an assay traceable to an isotope dilution mass spectrometry.

#### Screening and enrollment of participants

We aim to enroll 2000 HIV-positive individuals over a period of 2.5 years. Participants are recruited using various approaches, including use of study bulletins and brochures, provider referrals, and study informational “hotline”. All HIV-positive individuals receiving ambulatory care at the Moore Clinic are screened for enrollment at the time a patient attends an ambulatory clinic visit and provides permission to be approached by investigators, at the time a patient contacts study personnel directly, or at a separate screening visit at the Johns Hopkins Outpatient Clinical Research Unit.

#### Retention strategies

Retention of participants is critical to the successful completion of the study. Based on the data from the JHHCC [12], we anticipate that approximately 5% of participants will be lost to follow-up annually. To minimize participant drop-out, we have implemented several retention strategies. First, we have developed a study tracking system that maintains up-to-date contact information for the participant, secondary contact persons, and clinical providers. This tracking system also includes detailed information on scheduled and upcoming visit dates. Second, we ensure that participants are reminded of upcoming scheduled study visits via written letters and telephone calls. Third, participants receive compensation for each of the study visits they complete. In addition, the study links to registries, such as the U.S. Renal Data System and the National Death Index to ascertain outcomes on renal replacement therapy or kidney transplantation and vital status, respectively. For those participants also enrolled in the JHHCC, we will link with the JHHCC database for follow-up on clinical data. As part of our quality assurance and insurance practices, we monitor completion rates of participant contacts and study visits.

### Timeline of study visits during active follow-up

Individuals enrolled in the study will participate in a series of study visits for collection of data and biospecimens which are summarized in Table 1. After the baseline study visit, participants will return for annual ambulatory follow-up visits for the first 3 years of the study or until their first hospital admission during follow-up. Those who are hospitalized and who develop hospitalized clinical AKI will be identified in real-time by using computer-based programs linked to the Johns Hopkins electronic health records. Participants who are hospitalized undergo additional inpatient study visits within 24 h, 48 h, and 72 h of hospitalization, at the time of AKI diagnosis (if applicable), and at the time of hospital discharge. Following hospitalization, these individuals participate in two additional ambulatory in-person study visits at 3 and 12 months post-discharge and one telephone contact at 6 months post-discharge.

**Table 1:**
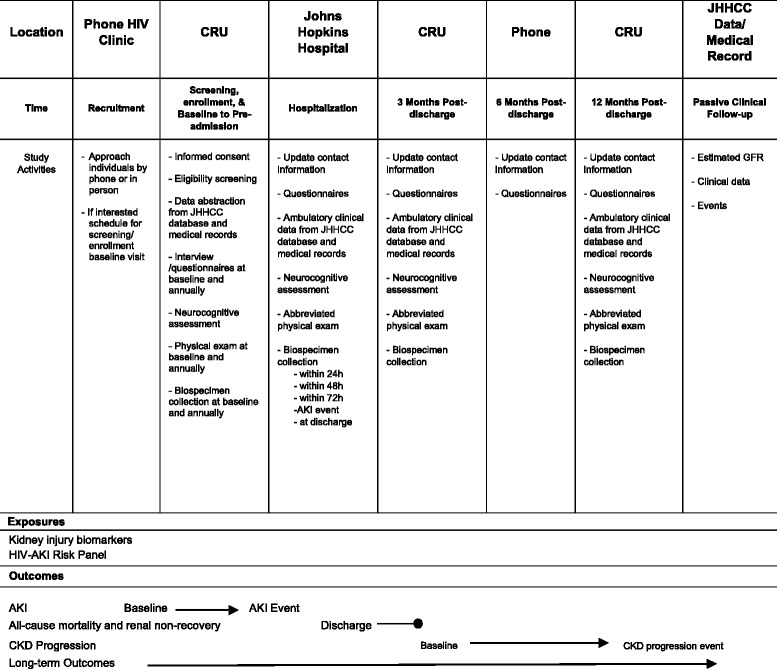
Overall study timeline and corresponding primary exposures and outcomes of interest

*JHHCC* Johns Hopkins HIV Clinical Cohort, *CRU* Clinical Research Unit, *GFR* Glomerular Filtration Rate, *AKI* Acute Kidney Injury, *CKD* Chronic Kidney Injury

### Data collection procedures

Data are collected through study visits, participants’ electronic health records, JHHCC database, and national registries (Table 2). During ambulatory study visits, detailed data are collected on participants’ contact information, health provider information, socio-demographics, behavioral history, medical and family history and current as well as prior medication use, including antiretroviral therapy and over-the counter medications. In addition, participants undergo standardized resting blood pressure and anthropometric measurements (height and weight), short assessments of health literacy (SAHL-E) [16] and neurocognitive assessments using Part A and B Trail Making Tests [17].

**Table 2:** Schedule for data collection and participant contact for the PARIS Study

	Baseline Visit	Year 1 ambulatory follow -up	Year 2 ambulatory follow -up	Year 3 ambulatory follow -up	Hospitalization within 24 h	Hospitalization within 48 h	Hospitalization within 72 h	Hospitalization AKI	Hospitalization at discharge	3 months post discharge	6 months post discharge	12 months post discharge	
	V0	V1	V2	V3	I0	I1	I2	I3	I4	P1	P2	P3	Passive follow-up
Eligibility screen	x												
Informed consent	x												
Contact information	x	x	x	x					x	x	x	x	
Provider contact	x	x	x	x									
Demographic data	x	x	x	x									
Baseline Medical history	x												
Ambulatory follow-up medical history		x	x	x									
Post-hospitalization medical history										x		x	
Family history	x												
Baseline social history	x												
Ambulatory follow-up social history		x	x	x									
Baseline medication use	x												
Follow-up medication use		x	x	x						x	x	x	
Study visit med list	x	x	x	x	x					x		x	
Ambulatory physical exam (BP, weight, height, waist circumference and hip circumference)	x	x	x	x	x					x		x	
Admission physical exam (BP and weight)					x								
Short assessment health literacy	x												
Trail-making Test Part A	x	x	x	x						x		x	
Trail-making Test Part B	x	x	x	x						x		x	
Post-hospitalization phone interview											x		
Hospitalization discharge medical records and summary													x
ESRD and kidney transplantation													x
Clinic visit medical records													x
Vital status													x
Stored blood	x	x	x	x	x	x	x	x	x	x		x	
PAXgene RNA	x	x	x	x						x		x	
Stored urine	x	x	x	x	x	x	x	x	x	x		x	
Comprehensive/ basic metabolic panel	x	x	x	x	x	x		x	x	x		x	x
HIV disease stage markers (HIV RNA level and CD4 cell count)	x	x	x	x	x					x		x	x
CBC	x	x	x	x	x	x		x	x	x		x	x
Urinalysis	x	x	x	x	x	x	x	x	x	x		x	x
Drug screen					x								x

V0: Baseline visit; V1: Year 1 ambulatory follow-up; V2: Year 2 ambulatory follow-up; V3: Year 3 ambulatory follow-up; I0: Hospitalization within 24 h.; I1: Hospitalization within 48 h.; I2: Hospitalization within 72 h.; I3: Hospitalization at AKI event; I4: Hospitalization at discharge; P1: 3 months post-discharge visit; P2: 6 months post-discharge visit; P3: 12 months post-discharge visit

In order to account for potential confounders, detailed data on ambulatory clinic visits and hospitalizations are also collected through the participants’ electronic health records (Table 3). These data include pre-existing kidney disease, other co-morbid conditions, and hospitalization-related factors. Specifically, the presence of pre-existing chronic kidney disease based on albuminuria, proteinuria and/ or eGFR is ascertained. Moreover, as hypertension and degree of blood pressure control are relevant to AKI risk and eGFR decline [18, 19], we assess for baseline hypertension and longitudinal blood pressures. Comprehensive, accurate ascertainment of medication use is critical for drug-related impact on AKI risk and subsequent kidney function decline. Therefore, we gather detailed data on use of antiretroviral medications, non-steroidal anti-inflammatory drugs (NSAIDs), antibiotics, and renin-angiotensin system (RAS)-antagonists (e.g. ACE-inhibitors). In addition, we obtain hospitalization-related data, including reason for admission, discharge diagnoses, procedures and if applicable, clinical AKI severity.

**Table 3:** Potential confounders of the association between biomarkers and outcomes

Potential confounders		Outcomes	
		Incident hospitalized AKI	Subsequent kidney function
Socio demographic	Age	Baseline	Baseline
	Race	Baseline	Baseline
	Gender	Baseline	Baseline
	Injection drug use	Baseline	Time-varying post-discharge
Baseline CKD	Albuminuria	Baseline	Baseline
	eGFR	Baseline	Baseline
Chronic Co-morbid Conditions	HCV co-infection	Baseline history	Baseline history
	Diabetes Mellitus	Baseline history	Baseline history
	Hypertension	Baseline history, ambulatory systolic & diastolic blood pressure	Baseline history, ambulatory systolic & diastolic blood pressure
	Cardiovascular disease	Baseline history	Baseline history
HIV Disease Stage	AIDS history	Time-updated absorbent state	Time-updated absorbent state
	CD4+ cell count	Time-varying	Time-varying
	HIV-1 RNA level	Time-varying	Time-varying
Medication Exposure	Antiretroviral drugs (evaluated as HAART, ART classes and individual drugs)	Cumulative	Cumulative post-discharge
	NSAIDs	Cumulative	Cumulative post-discharge
	RAS-antagonist	Cumulative	Cumulative post-discharge
Hospitalization-related factors	Antibiotic (by class)	Cumulative	In-patient exposure
	IV contrast	—	In-patient exposure
	AKI	—	AKI stage; peak serum creatinine
	Intensive care unit stay	—	In-patient exposure
	Duration of hospitalization	—	
	Primary discharge diagnosis	—	Categorized: Cardiovascular, infectious, respiratory, gastrointestinal, cancer, other
Anthropometric measurements	Weight	Baseline	Baseline
	Height	Baseline	Baseline
	Waist circumference	Baseline	Baseline
	Hip circumference	Baseline	Baseline

*AKI* Acute Kidney Injury, *CKD* Chronic Kidney Disease, *eGFR* estimated Glomerular Filtration Rate; *HCV* Hepatitis C Virus, *AIDS* acquired Immune Deficiency Syndrome, *CD4* Cluster of Differentiation 4; Human Immunodeficiency Virus; *HAART* Highly Active Antiretroviral Therapy, *ART* Antiretroviral Therapy; HIV: *NSAIDs* Nonsteroidal Anti-inflammatory Drugs, *RAS* Renin-Angiotensin System, *IV* Intra-Venous

We will enrich the PARIS data collection through annual linkage with the JHHCC database for participants who are also enrolled in the JHHCC. Similar to PARIS, data are collected through patient interviews, electronic patient records, other electronic sources of ambulatory/ inpatient services, and records from other facilities. These data sources are routinely captured per clinical protocol and include hospitalizations within the Johns Hopkins Health System and other facilities in Baltimore, MD, USA and visits to any of the outpatient primary care and specialty services and emergency department. Clinical data collected by the JHHCC include comprehensive data on clinical chemistry laboratory, radiological and pathological results, pharmacy use (including prescriptions and refills), and healthcare utilization. Of note, more than 95% of laboratory data are collected electronically through direct linkage to Johns Hopkins databases and outside laboratories. The JHHCC also links to vital statistics databases and the USRDS.

### Biospecimen collection and repository

As part of PARIS procedures, we collect biospecimens during the baseline and annual ambulatory study visits, hospitalizations, and post-hospitalization study visits as summarized in Table 2. Blood specimens are processed for serum, plasma and whole blood. We also utilize specialized collection and storage tubes for preservation of RNA. As handling and storage of urine specimens influence measurement of urine biomarkers [20], urine specimens are immediately refrigerated at 4 **°**C until laboratory processing within 2 h of collection. Urine specimens are subsequently processed to yield urine pellets, urine supernatant and uncentrifuged urine samples. All biospecimens are aliquoted and stored at −80 **°**C. For quality assurance and insurance, the date and timing of biospecimen collections and processing are recorded and monitored.

### Laboratory measurements

#### Method of serum creatinine measurement and GFR estimation

Standardized serum creatinine will be measured locally using an enzymatic method traceable to IDMS. This method has less measurement variability compared alkaline picrate assays for creatinine [21]. The serum creatinine-based CKD-EPI equation [15] will be used to estimate GFR.

#### Methods for urine biomarker measurement

To assess hospitalized AKI accurately, baseline assessment of kidney function and injury are essential. To determine the association of ambulatory kidney damage with incident hospitalized clinical AKI and progressive kidney disease after AKI and to investigate the prevalence of subclinical and clinical AKI among hospitalized HIV-positive individuals and their associations with progressive kidney disease after hospitalization, individual urine biomarkers of kidney injury will be measured using commercially available enzyme-linked immunosorbent assay (ELISA).

To develop a prognostic panel of complementary urine biomarkers that could be feasibly integrated into clinical practice, we will utilize results from single biomarkers to inform selection of an array of urine biomarkers which we will develop into a multiplex HIV-AKI Risk Panel based on the solid array-based method of Meso Scale Discovery array (Meso Scale Diagnostics, LLC., Rockville, MD, USA). Compared to readers for ELISAs, the electrochemiluminescence reader (MESO SECTOR) is several-fold more sensitive and covers a much broader range of biomarker concentration [22, 23]. Moreover, the electrochemiluminescence reader yields results more rapidly compared to those for ELISAs.

### Outcomes of interest

The outcomes of interest include hospitalized clinical AKI, hospitalized subclinical AKI, the composite of renal non-recovery and all-cause mortality at 3 months post-discharge, and kidney disease progression after 3-months post-discharge. Hospitalized clinical AKI and its severity will be defined using serum creatinine thresholds endorsed by the Kidney Disease: Improving Global Outcomes (KDIGO) guidelines on AKI [24] (Table 4). Hospitalized subclinical AKI is defined as an increase in urine biomarker level of 25% or greater from the most recent ambulatory level, among those who do not meet the criteria for clinical AKI. We will also evaluate alternative definitions, defining subclinical AKI among those who do not meet criteria for clinical AKI as: 1) a urine biomarker level above the top quartile of those without AKI; or 2) ≥4 biomarkers with levels ≥25% above the most recent ambulatory levels. Because surveillance for subclinical AKI is completely novel in this setting, we will pursue an iterative analytical approach.

**Table 4:** Acute kidney injury definition and staging based on serum creatinine

Definition	• Increase =0.3 mg/dl within 48 h OR • =1.5× baseline which is known OR • Presumed to have occurred within the prior 7 days
Stage	
1	• 1.5–1.9× baseline OR =0.3 mg/dl increase
2	• 2–2.9× baseline
3	• Increase to =4 mg/dl OR • Initiation of renal replacement therapy

For the composite outcome of renal non-recovery at 3 months and all-cause mortality among participants who are hospitalized and develop clinical AKI, renal non-recovery is defined as >25% decrease in eGFR at 3 months post-discharge from the most recent ambulatory level. Deaths are ascertained through abstraction of medical records, including those from outside institutions and death certificates. The vital status of participants who have not presented to the PARIS study visits or HIV Clinic and were last known to be living is checked annually through various vital statistic sources, including the National Death Index, Maryland Vital Records, and Social Security Death Files. Outcomes assessment at 3 months following hospitalization has been recognized as a potentially meaningful time point by the National Institute of Diabetes and Digestive and Kidney Diseases and the Food and Drug Administration for the assessment of urine biomarkers [25].

Among hospitalized participants with eGFRs ≥15 ml/min/1.73 m^2^ at 3 months post-discharge, the outcome of kidney disease progression will be evaluated. Post-discharge eGFR will be assessed using repeated estimates of GFR at 3 months, 12 months and up to 4 years of active follow-up. For eGFRs beyond 12 months, we will use the eGFR closest to the 3 months post-discharge date (i.e. annualized eGFR). This approach allows unbiased ascertainment of post-discharge eGFR; however, we will also conduct sensitivity analyses in which we use all available eGFRs after 3 months post-discharge. Kidney disease progression will be defined by the development of >30% decline in eGFR or progression to stage 5 CKD (eGFR <15 mL/min/1.73 m^2^, or ESRD) during longitudinal follow-up [26]. ESRD events will be ascertained by medical record abstraction and annual linkage to the U.S. Renal Data System, and JHHCC database.

### Sample size and power estimations

To determine the cohort sample size, we assumed 80% power and a two-tailed alpha of 0.05. Where possible, the standard deviations (SD) for biomarkers were taken from published literature or observations in TRIBE- AKI Consortium [4]. We estimated the effect size for the nested case-cohort analyses by simulations using the Nested Cohort package in R (R Foundation for Statistical Computing). Other calculations were conducted using SAS 9.3 (SAS Institute, Inc.). We estimated the number of events based on observations in the JHHCC during a 2-year period, with 700 hospitalizations and 229 clinical AKI events. Of those with clinical AKI, we estimated that 25% will have renal non-recovery and 6% will die within the first 3 months of discharge. Of persons who develop clinical AKI and survive beyond 3 months post-discharge, 40% will experience kidney disease progression during follow-up. Among those without clinical AKI, 25% will develop kidney disease progression.

For the nested case-cohort approach to evaluate the association of ambulatory urine biomarkers of kidney injury with hospitalized clinical AKI, we have sufficient power to detect a hazard ratio of 1.24 per 1-SD difference in urine biomarker level. For the composite outcome of renal non-recovery at 3 months and all-cause mortality post-discharge, there is sufficient power to detect an odds ratio of 1.65 per 1-SD difference in urine biomarker level. In addition, we have sufficient power to detect a relative hazard of 1.38 per 1-SD difference in urine biomarker level for the outcome of kidney disease progression. When comparing the risk of kidney disease progression among participants who develop subclinical AKI to those who do not develop clinical AKI, there is sufficient power to detect a hazard ratio of 1.26. For the development and validation of prognostic models, effect sizes exceeding the estimated minimum detectable effects are needed for consequential risk stratification [27], and we will be well-powered to detect the required effect sizes. Lastly, the number of events for each outcome relative to the number of candidate predictors is within recommendations for the development of prediction models [28].

## Discussion

In this paper, we highlighted the need for studies to address the excess burden of AKI in the HIV-positive population. The current model of AKI is incomplete since it is defined solely based on changes in kidney function estimates as assessed by serum creatinine. Previous and current studies of AKI have also frequently lacked baseline ambulatory measurements of creatinine levels, leading to AKI misclassifications and biased results. Most importantly, the current structure of HIV clinical research studies do not adequately bridge the ambulatory and inpatient setting. In addition, few studies addressing AKI have included sufficient numbers of African Americans despite their heightened risk of AKI and health consequences. Current methods to measure urine biomarkers are not feasible for clinical application.

PARIS addresses these key gaps in knowledge and will advance the progress towards implementation of urine biomarkers of kidney injury in clinical practice. This study will serve as an important resource for future studies of AKI by establishing a repository with both ambulatory and inpatient biospecimens which are currently unavailable in existing HIV-positive cohorts. Upon completion of this study, the resulting predictive model which will incorporate results from the multiplex HIV-AKI Risk Panel could be evaluated as a screening tool for trials comparing management strategies for AKI, and the Panel could serve as a surrogate endpoint for early phase AKI therapeutics.

Our study comes with some limitations. First, how best to define subclinical AKI remains unclear. We will explore alternative definitions of subclinical AKI and will evaluate how these definitions correlate with clinical variables and influence our estimates. This approach will enable us to examine the robustness of our findings and will inform future research in subclinical AKI. Second, this is a single center cohort enriched with African American patients. While we view this as a strength of the study, the composition of our study population may ultimately limit the extrapolation of our findings to the general HIV-positive population. Third, we cannot externally validate our predictive model due to the lack of paired ambulatory and inpatient urine samples required for studying AKI in existing clinical HIV cohorts. To address some of these limitations, we envision a future clinical trial to assess the utility of the HIV-AKI prognostic model compared to standard of care in reducing time to recognition and sequelae of AKI in the HIV-positive population.

In conclusion, this study will serve as an important resource for future studies of AKI by establishing a repository with both ambulatory and inpatient biospecimens that is currently lacking in existing HIV clinical cohorts and studies of AKI. Upon completion of this study, the resulting prognostic model which incorporates results from the multiplex HIV-AKI Risk Panel could be evaluated as a screening tool in trials comparing early management of AKI or intensive management after AKI with current standards of care, and the Panel could serve as a surrogate endpoint for early phase therapeutic candidates for AKI.

## Abbreviations

AKI: Acute kidney injury
CKD: Chronic kidney disease
eGFR: estimated glomerular filtration rate
ELISA: Enzyme-linked immunosorbent assay
ESRD: End-stage renal disease
HIV: Human immunodeficiency virus
IL-18: Interleukin-18
JHHCC: Johns Hopkins HIV Clinic Cohort
KIM-1: Kidney injury marker-1
NGAL: Neutrophil gelatinase-associated lipocalin
PARIS: Predictors of Acute Renal Injury Study
SAHL-E: Short assessments of health literacy
SD: Standard deviation
TRIBE-AKI: Translational Research Investigating Biomarker Endpoints in Acute Kidney Injury
